# Comparing the effectiveness and lubricity of a novel Shea lubricant to 2% lidocaine gel for digital rectal examination: a randomized non-inferiority trial

**DOI:** 10.1038/s41598-023-31555-2

**Published:** 2023-03-22

**Authors:** Kekeli Kodjo Adanu, Davidson Iroko, Kokou Amegan-Aho, David Adedia, Orish Verner Ndudiri, Mahamudu Ayamba Ali, Mawuenyo Attawa Oyortey, Jacques Kpodonu

**Affiliations:** 1 Department of Surgery, School of Medicine, University of Health and Allied Sciences, Ho, Ghana; 2 Department of Anaesthesia and Critical Care, School of Medicine, University of Health and Allied Sciences, Ho, Ghana; 3 Department of Pediatrics, School of Medicine, University of Health and Allied Sciences, Ho, Ghana; 4 Department of Basic Sciences, School of Basic and Biomedical Sciences, University of Health and Allied Sciences, Ho, Ghana; 5 Department of Microbiology, School of Medicine, University of Health and Allied Sciences, Ho, Ghana; 6 Faculty of Surgery, Harvard Medical School, Boston, USA

**Keywords:** Drug discovery, Medical research, Urology

## Abstract

This study compared the level of lubricity and pain reduction of a novel shea lubricant to 2% lidocaine gel during digital rectal examination (DRE). Our research group performed a 9-week single-blind non-inferiority trial at the Ho Teaching Hospital involving 153 patients. The primary outcome measure was the mean pain difference during the procedure using a Visual Analogue Scale. 75 and 78 patients were randomized to the shea lubricant and 2% lidocaine gel groups respectively. The analysis considered the per-protocol population. The mean pain difference at endpoint was Δ − 0.01. The 95% lower confidence interval was a -0.595 difference in means, above the non-inferiority (NI) limit of − 0.720, thus establishing non-inferiority (Δ − 0.01, 95% CI − 0.59 to 0.57, NI − 0.72). With secondary outcome measures, perianal pruritus (p = 0.728), discomfort (p = 0.446), bowel urgency (p = 0.077) and urinary urgency (p = 0.841) were similar during the procedure. Shea lubricant had better lubricity and ease of use (p = 0.002). While the novel shea lubricant achieved similar level of pain reduction as obtained with 2% lidocaine gel, it had better ease of performance and lubricity.

## Introduction

Medical lubricants are valuable tools and are important in the care process. Regulations require that lubricants are discreet, non-toxic and effective. In choosing a lubricant for a surgical procedure, care must be taken to ensure it is fit for purpose. It should be physiologically inert and stable at room and extreme pressures^1^. Several substances have been used as lubricants including glycerol, emulsions, propylene glycol, oils and gels. Lubricant gels in particular have been found to be conducive because they can remain in-situ without dripping. They ought to be sterile but even with the strictest aseptic precautions, infection rates as high as 30% have been reported^2^. More recent studies have shown that these lubricants can be intrinsically contaminated from the source and are a potential cause of nosocomial infection^3,4^. Procedures such as cystoscopy, endoscopy, nasolaryngoscopy and passage of nasogastric tubes, urethral catheterization and digital rectal examination (DRE) involve the use of lubricants. Digital rectal examination is a simple but useful procedure for the examination of several medical conditions. It is used for the assessment of anal and rectal tumors, anal fissures, prostate lesions, lower gastrointestinal hemorrhage, bowel lesions resulting from trauma and other medical ailments. Lubricants widely used include the 2% lidocaine gel and the K-Y gel.

Lidocaine gel is commonly used for medical procedures in Ghana. However, it is not always available because of its prohibitive cost and procurement delays. Patients sometimes had to buy them at exorbitant prices from private pharmacies for procedures to be carried out, in addition, physicians may at times, resort to the usage of non-appropriate products to get around the situation. The use of inappropriate materials such as soapy water, chlorhexidine solutions and plain water have led to considerable discomfort and pain to patients.

Shea butter is a widely available product obtained from the shea tree (*Vitellaria paradoxa)* which is indigenous to sub-Saharan Africa, extending across some nineteen (19) countries^5^. The sweet pulp of the fruit is a good source of proteins, sugars, ascorbic acid and iron. The fat extracted from the shea kernel is the shea butter which has wide applications in the cosmetics, food, and medical industries. Shea butter is mainly composed of triglycerides to which anti-inflammatory and antioxidant characteristics are attributed hence its high demand in the cosmetic industry. It is also a staple cooking oil in the Sudanian savanna zone^5^. Lin et al.^6^ reported the anti-inflammatory effect of shea butter through its inhibition of cyclooxygenase pathways, hence its significant use in the treatment of dermatological ailments.

Shea butter, which is known for its anti-inflammatory and antioxidant effects and because of its oily constituent could make a good replacement in areas where lidocaine gel is not available. The crude shea butter is used as a lubricant for surgical procedures in some Ghanaian hospitals however there is no available data on its use and efficacy as a medical lubricant. The main lubricants found in K-Y gel are glycerin and cellulose which are also found in the shea fruit^5^. Shea butter, which is locally produced, cheaper and readily available, when adequately processed and sterilized, may serve as a good non-inferior substitute to the aforementioned products due to its similar constituents. Our vision is to transform this raw material into a refined product that can be used for procedures in Ghanaian hospitals and other medical facilities worldwide provided it met the required safety and biomedical standards.

This novel shea lubricant is constituted from refined shea olein. Added to this is an acrylic acid polymer, an emulsifier (propylene glycol) and preservatives (methyl paraben and propyl paraben) to achieve a unique blend. It is sterilized by heating and stored under room temperature conditions away from direct sunshine. The product has also been subjected to physiochemical and microbiological analysis and stored in a collapsible sealed plastic tube.

Cost analysis also showed that lidocaine gel is more expensive than plain lubricants. Chen and coworkers^7^ reported that the cost of lidocaine gel is about three times that of plain gel. The cost per 100 ml of lidocaine gel and plain lubricant was 220 and 66.6 Taiwan dollars respectively (in 2005). Mcfarlene and colleagues^8^ also confirmed the relatively high cost of lidocaine gel at their institution. It emerged that cost savings could be more than GBP 3876 (USD 5000) per year if lidocaine gel was eliminated from cystoscopy examinations. A 30 g tube of lidocaine gel on the Ghanaian market cost an average of USD 5.00. These compelling reasons obligated us to investigate further. Our hypothesis seeks to determine whether oil-based lubricants such as shea butter could be as effective as the standard reference of care, 2% lidocaine gel.

Lidocaine gel remains the reference standard for most medical procedures as enumerated by several trials and meta-analyses^9–12^. No previous trial has compared a shea lubricant to 2% lidocaine gel. Our choice of a non-inferiority design was based on the expectation that the non-inferiority of the Shea lubricant to 2% lidocaine gel will tip the scale in favor of Shea lubricant due to the accessibility, availability and pricing of the raw material for its production in Ghana.

In this randomized controlled, single-blind non-inferiority trial, we compare a novel shea lubricant to 2% lidocaine gel, in terms of its effectiveness in reducing anal pain, discomfort, peri-anal pruritus, urinary and bowel urgency; and also assess the ease of use of the lubricants by clinicians during digital rectal examination.

### Hypothesis

Our hypothesis is to test whether the shea lubricant can achieve the same level of pain reduction as obtained with the use of 2% lidocaine gel for digital rectal examination, at a better ease of performing the procedure. The confirmation of non-inferiority involved the pre-specification of a mean pain difference for shea lubricant as compared to 2% lidocaine gel below a predefined margin, based on a relative measure.

Primary outcome measures—mean difference in pain perception during the procedure with a non-inferiority limit set at − 0.72.

Secondary outcome measures—differences in the perception of discomfort, peri-anal pruritus, bowel and urinary urgency during the procedure using Likert scales. Secondly, to assess the ease of use of the lubricants by trial doctors.

## Materials and methods

This was a single-blind, randomized non-inferiority trial that compared 2% lidocaine gel to a shea lubricant, eliciting patients’ response to anal pain, discomfort, peri-anal pruritus, bowel and urinary urgency and ease of use of the lubricants by clinicians upon performing a digital rectal examination. All patients between 18 and 80 years for whom a digital rectal examination was indicated, could understand the survey process and consented to participate were included in the study. Those who were too ill to communicate and/or consent, and those with painful anal conditions such as thrombosed hemorrhoids, anal fissures, infiltrating anorectal cancers and anal strictures were excluded. Patients were recruited from 15th June to 19th August 2021.

### Study setting

This study was conducted at the surgical, urological and emergency units of the Ho Teaching Hospital which is the foremost referral facility in the Volta Region and attends to an average of 170,000 patients annually.

### Ethical considerations and trial registration

The trial was approved by the University of Health and Allied Sciences, Research Ethics Committee with protocol number UHAS-REC A. 2(4) 20 -2. It has been registered with the Pan African Clinical Trials Registry on 18/11/2020 with the unique identification number PACTR202011687956222 and the Food and Drugs Authority (FDA) Ghana Clinical Trials Registry with certificate number FDA/CT/217. The investigational product (shea lubricant) underwent physiochemical and microbiological analysis at the Ghana Standards Authority. The certificate of analysis (COA) of the control lubricant was similarly verified by FDA, Ghana. The trial was conducted according to the declaration of Helsinki and trial participants gave written informed consent before enrollment. The study protocol further outlines the rationale, design and methods of the trial^13^.

### Sampling

To estimate the sample size, we considered the standard deviations for pain perception after catheterization from a previous study done by Stav et al.^14^. We chose this study because it compared an oil-based lubricant to lidocaine gel, which is the intent of the present study. To determine the non-inferiority limit, we relied on a systematic review and meta-analysis by Hong et al.^15^ where the mean difference for pain was estimated at − 0.96 (95% confidence interval, − 1.43 to − 0.49) after including sixteen randomized trials. Thus non-inferiority limit was set at − 0.72 which is a 50% discount of the lower limit of the 95% confidence interval (− 1.43) of the mean pain reduction effect of the control lubricant (2% lidocaine gel); as shown in the literature^16^. An attempt to ensure that the shea lubricant preserved at least half the effect of 2% lidocaine gel. The trial was therefore designed to randomly allocate 153 patients with 90% certainty (power), assuming that the lower limit of the 95% confidence interval was within a prespecified boundary of − 0.72 in mean pain perception difference using the Visual Analogue Scale, with a 1:1 allocation ratio and ${\mu }_{1}-{\mu }_{2}$= 0 for non-inferiority trials, as shown in the literature^17,18^. Our trial participants were similar to participants in the randomized trial by Goldfisher et al.^9^ and Siderias et al.^10^ where the control lubricant (2% lidocaine gel) was investigated.

### Sampling procedure

There were two treatment arms, Shea Lubricant (A) and 2% lidocaine gel (B). A clinical trial randomization software (National Cancer Institute Clinical Trial Randomization tool) was employed to allocate patients to the two groups using a 1:1 allocation ratio. Patients assigned odd numbers were randomized to Group A and those assigned an even number, to Group B. The process continued till the required number of participants was obtained in the two groups. The examination procedure was explained after informed consent was sought from each patient. Participants were assured of their privacy, confidentiality and their rights to opt out of the trial at any point in time without affecting access to quality care. Structured questionnaires were administered before, immediately and 30 min after the procedure to assess pain perception, peri-anal discomfort and pruritus, and urinary and bowel urgency. The questionnaire also evaluated trial doctors’ assessment of ‘ease of use’ of the lubricants.

### Trial process

In all, two trial nurses independent of the study and eight (8) trial doctors were recruited. To ensure quality control, reliability and reproducibility, the trial recruits were trained in data collection, trial protocols and procedures, seeking informed consent and safe disposal of waste. The investigational products were stored at the hospital’s pharmacy under ambient conditions. The lubricants were prepared by the independent trial nurses and delivered in marked 5 ml—syringes labelled A and B to trial doctors. The control lubricant (Optilube active gel) has similar constituents to the reference lubricant used in previous trials^10,11^.

All DRE examinations were done in the presence of a chaperone due to the sensitivity of the procedure. After explaining the procedure and seeking consent, participants were asked to undress including undergarments in privacy. A gown was used to preserve modesty till the procedure was performed. Patients were made to lie on a couch in the left lateral position facing away from the examiner with the legs drawn up towards the chest. The examiner puts on sterile gloves and the examination starts with the inspection of the natal clefts (the groove between the buttocks), the anus and the perianal skin for lesions. Any lesion that constitutes a contraindication (refer to inclusion/exclusion criteria) led to the abandonment of the procedure. The patient is then asked to bear down, observing for lesions. The examiner lubricates the examining finger with 5 mls of the selected lubricant. At this stage, the examiner alerts the patient about the commencement of the procedure. The examiner gently pats the buttocks and applies gentle pressure at the anal margin to enter the anus and the rectum. The anal tone is determined by asking the patients to “squeeze” on the examining finger and release it. The finger is then directed posteriorly following the curve of the sacrum observing for any lesions, tenderness, tightness and mobility of the rectal mucosa. The finger is then swept anteriorly in a clockwise direction to examine the prostate in men and the pouch of douglas in women. The examination is completed by inspecting the examining finger for the nature of fecal matter, streaks of blood or any discharge.

### Stopping rules

Our stopping criteria were based on three ethical principles- safety, benefit or futility of the trial. Development of serious adverse events such as anaphylactic shock, urticaria, wheezing attacks or any other form of serious adverse drug reaction will trigger discontinuation. Our patients have been reviewed over the period and no adverse reaction has been observed.

### Methods of randomization and data collection

The allocation sequence was concealed from trial doctors and nurses and only revealed to the trial nurse upon the recruitment of a participant while keeping the trial doctor in oblivion. The sequence was generated by an investigator assigned for that purpose whilst the trial nurses enrolled, assigned participants to an intervention and also assessed outcomes. Study participants were blinded to the interventions. However, since the consistency of the products was distinguishable the trial doctors could not be blinded. The study used a quantitative data collection approach. A structured questionnaire consisted of three sections (1) socio-demographic characteristics, (2) patients’ perception, (3) ease of use of the lubricants. Patients’ perception of anal pain was evaluated with a Visual Analogue scale which rated pain from the most excruciating (10) to no pain (0). Peri-anal discomfort, pruritus, bowel and urinary urgency were assessed using 4-dimension Likert scales to each of the questions. Similarly, a 5-dimension Likert scale determined the ease of use of lubricants by clinicians. Questionnaires were administered through face-to-face interviews after written informed consent had been obtained with the privacy, confidentiality and security of patients’ data assured. Quality control measures instituted prior to data collection include (1) training of research assistants, trial doctors and nurses on data collection and informed consent. (2) Error correction. (3) Appropriate storage of investigational products. The data collected was entered daily and validated. The authors vouch for the accuracy of data and the fidelity of the study to the protocol.

### Data analysis

A detailed summary of the analyses of patients’ perceptions undertaken in the study is provided in Table 1 below.

**Table 1:** Summary of analyses of patients’ perception.

Perception	Tool	Estimation approach
Anal pain	Visual Analogue Scale (VAS)	A VAS tool assessed the perception of pain felt by patients during the digital rectal examination. The scale rated pain from 0 (no pain) to 10 (worst imaginable pain). The mean score was determined by summing up the score in each group and dividing it by the number of participants in that group. The mean difference in pain scores was deduced and compared to the non-inferiority limit of − 0.72 to ascertain the inferiority or otherwise of the shea lubricant
Peri-anal discomfort and pruritus	Likert scale	To estimate these variables, a 4-dimension Likert scale was adopted (i.e., [1] Nil, [2] Mild, [3] Moderate, [4] Severe) in relation to the level of peri-anal discomfort and pruritus perceived by patients in the two arms. Mean scores in both groups were compared to determine if there was a difference in perception
Urinary and bowel urgency	Likert scale	The urinary and bowel urgency score was obtained from responses to 4-dimension Likert scales (i.e., [1] Nil, [2] Mild, [3] Moderate, [4] Severe) in relation to the perceptions of urinary and bowel urgency experienced by participants
Ease of use of Lubricants and Lubricity	Likert scale	Trial doctors assessed the ease of use of lubricants and lubricity using a 5-dimension Likert scale (i.e. [1] Effortless, [2] Easy, [3] Fair, [4] Difficult and [5] Very difficult). The scores achieved for each option are expressed as mean scores and in percentage terms. The proportions obtained for each dimension in the two groups are then compared

### Statistical analysis

The collated data was entered into Excel 2013 and exported to SPSS 25 and R 4.1.2 for analysis. . Mann Whitney test was used to compare two independent groups. Categorical variables were reported using frequency tables and charts while continuous variables were reported using means and standard deviations. The study endpoints were analyzed for the per-protocol population. A sensitivity analysis was then conducted for the intention-to-treat population. For the primary endpoint analysis, a 95% confidence interval with a two-sided 5% level of significance approach was employed. A p-value ≤ 0.05 is statistically significant, however to reduce the chance of type I error in multiple testing, a p-value ≤ 0.003 is considered significant after correcting the alpha using the Bonferonni approach (A multiple tests of 5 variables with 3 levels (0.05/15 = 0.003)). The non-inferiority test was performed for only the primary end point, the secondary endpoints were analyzed for superiority. All missing values were excluded from the analysis.

## Results

**Figure 1:**
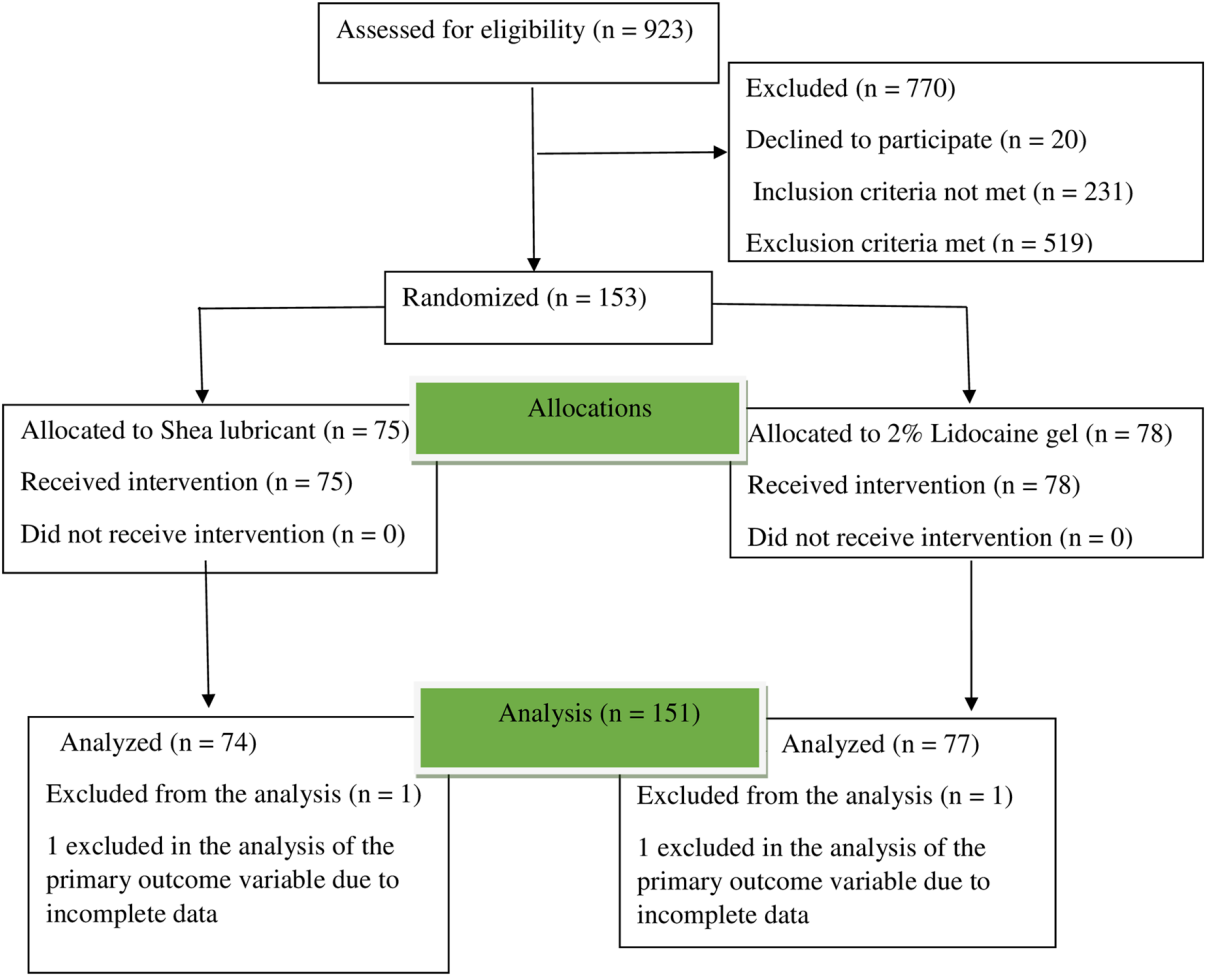
Summary of data collation and analysis.

Figure 1 summarizes participants’ flow, data collation and analysis throughout the trial. The baseline demographic characteristics (shea lubricant and 2% lidocaine gel) were fairly similar with no statistical difference in the age (p = 0.710) and marital status (p = 0.601) except for employment status (p = 0.040). 97% of participants were males. The overall mean age ± standard deviation was 63 ± 14.34 years.

Tables 2 and 3 summarized the rating of the lubricating effect of the shea lubricant versus the lidocaine gel as determined by the doctors who performed the DRE. Interestingly, neither the shea lubricant nor the lidocaine gel was rated as “poor” or “very poor” in terms of ease of use or lubricating effect. Generally, the shea lubricant had higher frequency and percentage for “easy” and “effortless” ratings compared to the lidocaine gel. Ease of use (U = 2044.0, p-value = 0.001) and lubricating effect (U = 2146.5, p-value = 0.002) for the shea lubricant were significantly better than the lidocaine gel.

**Table 2:** Lubricating effect of shea lubricant and lidocaine gel.

Variable	Shea lubricant	Lidocaine gel
	n (%)	n (%)
Ease of use		
Effortless	32 (42.7)	25 (32.0)
Easy	41 (54.7)	24 (30.8)
Fair	2 (2.7)	29 (37.2)
Lubricating effect		
Good	22 (29.3)	24 (30.8)
Very good	51 (68.0)	21 (26.9)
Neutral	2 (2.7)	33 (42.3)

n, frequency.

**Table 3:** Lubricating effect and Ease of use: mean scores.

Variable	Mean ± SD		p-value (U)
	2% Lidocaine gel	Shea lubricant	
Ease of use	2.05 ± 0.84	1.60 ± 0.55	0.001 (2044.0)
Lubricating Effect	2.12 ± 0.85	1.73 ± 0.50	0.002 (2146.5)

**Figure 2:**
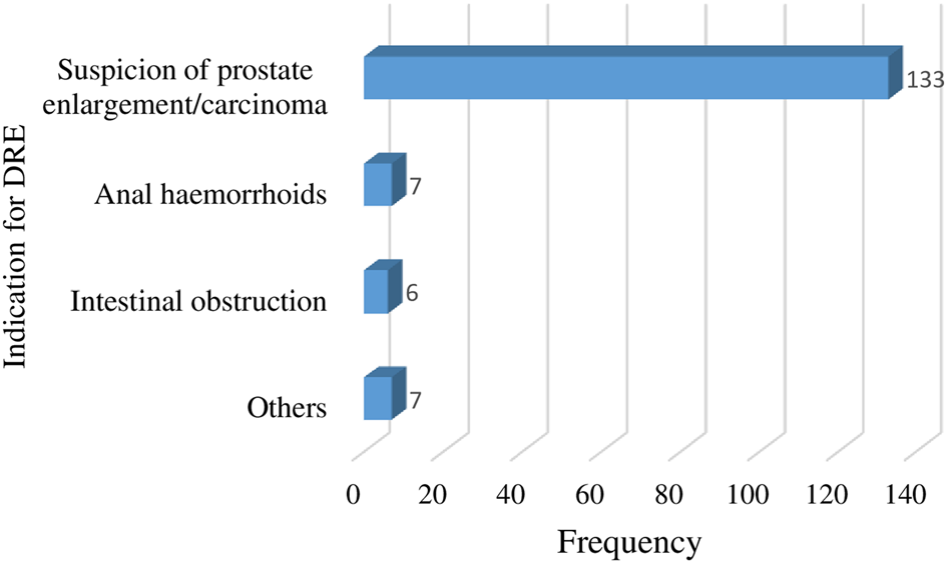
Indication for DRE.

**Figure 3:**
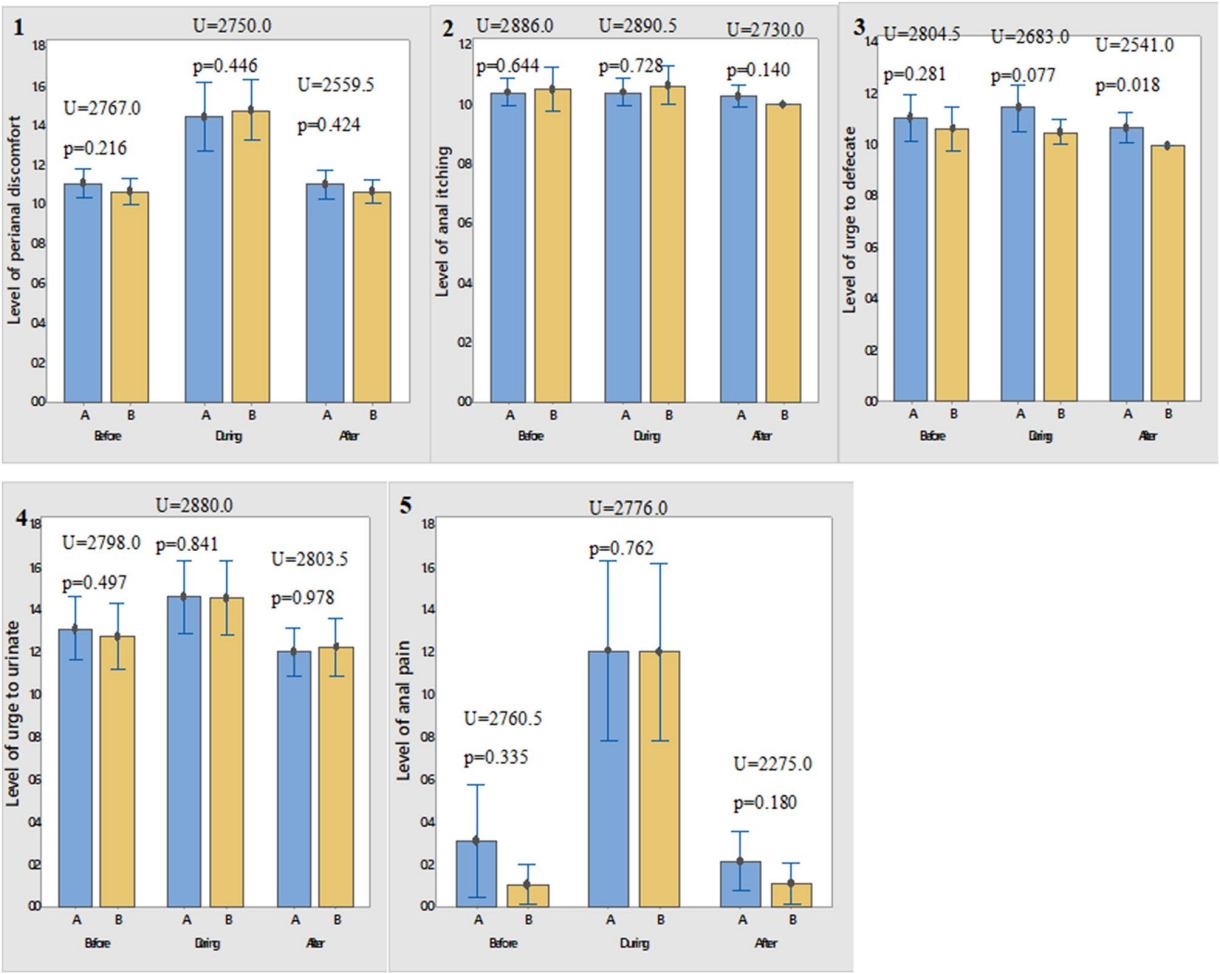
Differences in adverse effect before, during and 30 min after DRE for shea lubricant versus lidocaine gel.

As shown in Fig. 2, most of the participants were suspected of prostate enlargement/carcinoma (representing 86.9% of participants), the rest of the indications were anal haemorrhoids (4.6%), and intestinal obstruction (3.9%). The “other” indication were undifferentiated rectal carcinoma, urethral stricture, hydrocele, and testicular cancer At baseline, before the DRE was performed, participants had some level of the adverse effects being investigated for both lubricants. However, there was no significant difference in the level of perianal discomfort, itching in the anal region, urge to defecate (bowel urgency), urge to urinate (urinary urgency), and level of anal pain with both lubricants (Fig. 3).

**Figure 4:**
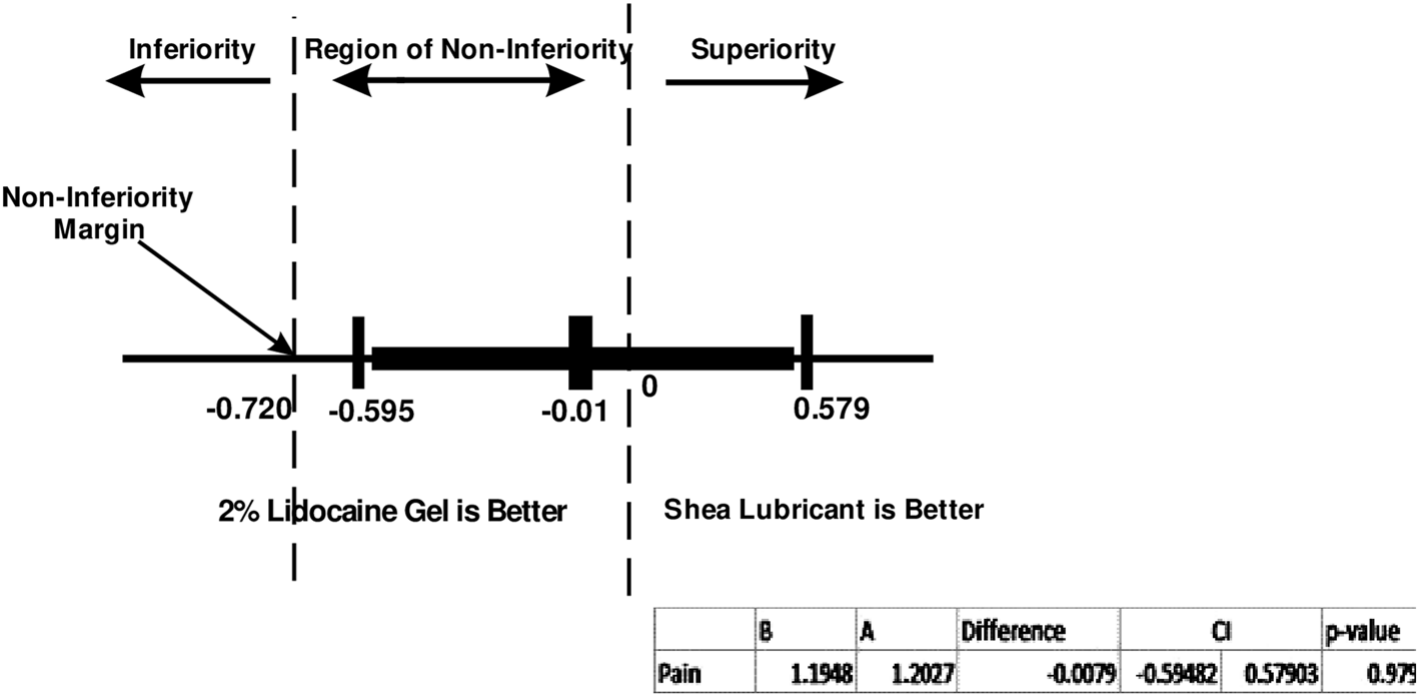
95% CI for mean difference of pain during procedure and Non-inferiority (per protocol population).

Figure 4 displays the confidence interval of the mean difference for pain (Δ − 0.01) and the non-inferiority margin (− 0.72). The lower limit of the confidence interval (− 0.595) is higher than the non-inferiority margin of − 0.72.

**Figure 5:**
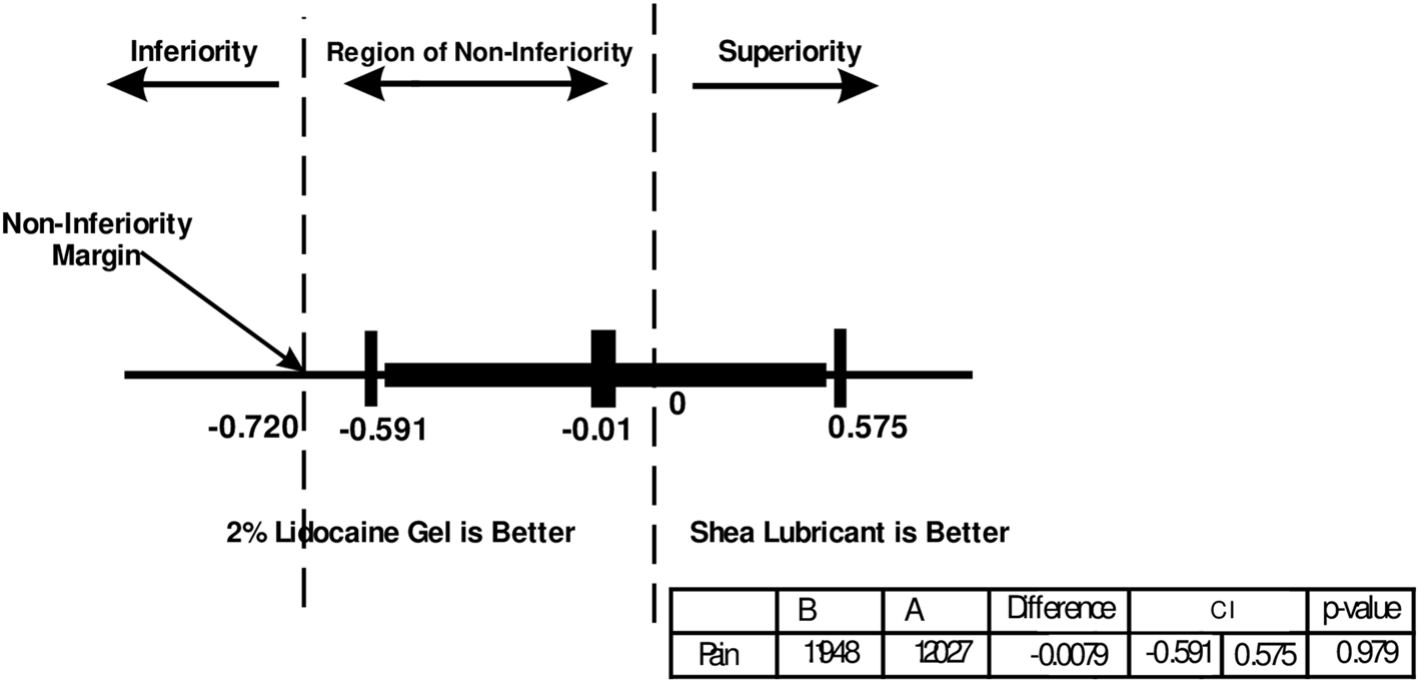
Sensitivity analysis to test primary outcome (intention-to-treat population).

Figure 5 shows sensitivity test based on the intention-to-treat population. The 95% confidence interval ranged from − 0.591 to 0.575.

## Discussion

This study was designed to ascertain the efficacy of the shea lubricant in achieving the same level of anal pain reduction as obtained with 2% lidocaine gel during the digital rectal examination. We found that the shea lubricant was just as effective as the 2% lidocaine gel in this non-inferiority trial and thus a good substitute for the control. Even more, the shea lubricant was rated as having a better level of lubricity (p = 0.002) and ease of use by the clinicians involved in the trial (p = 0.001). Secondary outcomes such as the level of perianal discomfort (p = 0.446), anal itching (p = 0.728), bowel urgency (p = 0.077), urinary urgency (p = 0.841) and anal pain (0.762) during the procedure were similar. The non-inferiority (NI) of the shea lubricant to the lidocaine gel for the mean difference in anal pain as set at the trial commencement was firmly established (Δ -0.01 95% CI − 0.595 to 0.579 Δ_NI_ − 0.72).

The baseline demographic characteristics (shea lubricant and 2% lidocaine gel) were fairly similar with no statistical difference in the age (p = 0.710) and marital status (p = 0.601) of participants except for employment status (p = 0.04). The main indications for DRE were prostate enlargement/carcinoma (87%), suspicion of rectal haemorrhoids (4.6%), intestinal obstruction (3.9%) and rectal carcinoma (0.7%). This is attributable to the fact that the study was carried out mainly within a surgical setting; these dynamics could have been different had it been carried out in another medical field. Marcias et al.^19^ in a separate study carried out in a trauma center identified gastrointestinal bleeding (32%) as the main indication for DRE, followed by abdominal pain (28%), chest pains (16%) and prostate complaints (13%).

To the best of our knowledge, this is the first study comparing the efficacy of 2% lidocaine gel to a vegetable oil-based lubricant for digital rectal examination. The mean pain scores evaluated using the Visual Analogue Scale were similar. This finding is consistent with results obtained by Carrion et al.^20^ and Chen et al.^7^ where 2% lidocaine gel was compared to plain lubricants for flexible cystoscopy. On the other hand, it was dissimilar to findings by Siderias et al.^10^ and Chan et al.^21^ where 2% lidocaine gel was found to have a better pain reduction effect than a plain lubricant during urethral catheterization. Interestingly, in another study^14^ where an oil-based lubricant-paraffin was compared to 2% lidocaine gel; as was the case in this trial, it emerged that paraffin was more efficient in reducing urethral pain during urodynamic analysis. A possible explanation for these findings as the authors postulated could be that liquid paraffin was a better lubricant than the aqueous-based lidocaine gel and that the lubrication characteristic of a lubricant could be more important than the mere presence of an anesthetic agent. This explanation may account for why the lubricity of the shea lubricant in this study was rated higher than 2% lidocaine gel (p = 0.002). In our considered view, the mean pain difference observed in Stav et al.^14^ was not replicated in this trial because the comparator parameter, DRE, unlike urodynamic studies elicited less pain. A procedure associated with more considerable pain could have discriminated between the two lubricants. Furthermore, from a clinical viewpoint, patients’ tolerability of a procedure is just as important as the perception of pain. The tolerability is dependent on the ease of performing the procedure. When trial doctors assessed the ease of performing rectal examinations, the shea lubricant was rated as either “easy” or “effortless in 97% of responses compared to 87% for 2% lidocaine gel. This difference was found to be significant (p = 0.001). This demonstrates that surgical procedures may be performed at a better ease using shea lubricant than lidocaine gel. The possibility of clinicians’ bias however cannot be overlooked since the lubricants were of different consistencies and could be distinguished. Our attempt to mitigate this bias by dyeing the lubricants was not successful due to the differential thickness of the shea lubricant. Future trials may explore innovative ways of mitigating this effect. Although not directly comparable, Chuah et al.^22^ in their report on pharyngeal anaesthesia comparing lidocaine spray and placebo during upper gastrointestinal endoscopy did not find any difference in the ease of passage and tolerability of the procedure. Meaning, the impact of lidocaine on the tolerability of medical procedures may be minimal.

The experiences of patients with regards to peri-anal discomfort, peri-anal itching, urinary urgency and bowel urgency were similar for the two groups before, during and 30 min after the procedure.. In a similar study on patients’ perceptions of pain and discomfort during DRE, Romero et al.^23^ found no difference in bowel and urinary urgency between the groups compared.

At the outset, the non-inferiority limit for anal pain perception was set at a mean between-group difference of − 0.72 based on a previous study^15^. Our trial achieved a mean difference of -0.01 showing that indeed, the shea lubricant was non-inferior to the control. The mean pain scores for shea lubricant and lidocaine gel were 1.20 and 1.19 respectively on the VAS. An analysis conducted on the intention-to-treat population to test the robustness of the primary outcome confirmed the non-inferiority of the shea lubricant (Δ − 0.01 95% CI − 0.591 to 0.575). In a crossover randomized trial, Chitale et al.^24^ found no difference in pain perceptions after 51 patients were randomized to receive either an anaesthetic gel or plain gel during cystoscopy. A non-inferiority test conducted showed that the difference in pain perception was insignificant. In contrast, a meta-analysis by Raskolnikov and associates^25^, where 12 randomized control trials were included suggested that anaesthetic agents achieved a better pain control than plain lubricants.

The findings from this study is important as it confirms the extent to which the shea lubricant can be a good non-inferior substitute to the control. Coupled with the fact that it can be produced at a competitive cost, it is advantageous to advocate for the regular use of the shea lubricant, especially in low-resource settings as it has similar pain reduction and complication rates as lidocaine gel. Mcfarlene et al.^8^ found that cost savings could be more than GBP 3876 (USD 5000) per year if lidocaine gel was eliminated from cystoscopy examinations Cost savings may also be observed in low-resource settings if lidocaine gel is eliminated. A 10 ml pre-filled syringe of 2% lidocaine gel is being sold for an average price of USD 5 on the Ghanaian market compared to the production cost of USD 0.71 for a 100 ml tube of the shea lubricant. To put this in context, the daily minimum wage in Ghana is approximately USD 1, meaning the cost of the lidocaine gel is about five times the minimum wage. It is prudent therefore to produce the shea lubricant on a larger scale due to its lower cost of production and accessibility to raw materials which in the long-term, will benefit the West African subregion due to the high economies of scale anticipated. It is further suggested that despite trade and sociocultural barriers a well-refined and standardized shea lubricant would compete favorably with lubricants elsewhere, just like how cosmetics from shea butter are well regarded worldwide. Nevertheless, there are still some pertinent issues that need to be addressed. How well will the shea lubricant compare to a plain lubricant, since the cost of production may be similar and how will it fare when used in other medical procedures? These concerns may be the objects of future investigations.

Although our findings are significant and generalizable, there were some constraints. Due to differences in the consistency of the lubricants, the trial doctors were not blinded even though attempts were made to mitigate its effect. Larger cohorts are required to further test the efficacy and long-term effects of the shea lubricant, especially in procedures such as urethral catheterization and endoscopy.

## Conclusion

Shea lubricant achieved similar level ofpain reduction as obtained with 2% lidocaine gel for digital rectal examination, at a better ease of performing the procedure. Similar complications were observed in the two groups. We will advocate for trials in larger cohorts to further test the efficacy of the shea lubricant in other procedures such as urethral catheterization.

## Data Availability

The datasets generated during and/or analysed during the current study are available in the figshare repository, 10.6084/m9.figshare.20363166. Further details can be found in data sharing statement.

## Data sharing statement

Will individual participant data be available (including data dictionaries?—Yes. What data in particular will be shared?—Individual participant data that underlie the results reported in this article after deidentification (text, tables, figures and appendices). What other documents will be available—Informed consent form, Questionnaire. When will data be available (start and end dates)?—Immediately after publication. No end dates. With whom?—Anyone who wishes to access the data. For what types of analyses?—To achieve aims in the approved proposal. By what mechanism will data be made available?—Data are available indefinitely at figshare repository, 10.6084/m9.figshare.20363166.
